# Low BACH2 Expression Predicts Adverse Outcome in Chronic Lymphocytic Leukaemia

**DOI:** 10.3390/cancers14010023

**Published:** 2021-12-21

**Authors:** Carmela Ciardullo, Katarzyna Szoltysek, Peixun Zhou, Monika Pietrowska, Lukasz Marczak, Elaine Willmore, Amir Enshaei, Anna Walaszczyk, Jia Yee Ho, Vikki Rand, Scott Marshall, Andrew G. Hall, Christine J. Harrison, Meera Soundararajan, Jeyanthy Eswaran

**Affiliations:** 1 Department of Applied Sciences, Faculty of Health and Life Sciences, Northumbria University, Newcastle upon Tyne NE1 8ST, UK; carmela86@gmail.com (C.C.); meera.soundararajan@northumbria.ac.uk (M.S.); 2 Translational & Clinical Research Institute, Newcastle University, Newcastle upon Tyne NE1 7RU, UK; k.s.szoltysek@prinsesmaximacentrum.nl (K.S.); elaine.willmore@newcastle.ac.uk (E.W.); amir.enshaei@ncl.ac.uk (A.E.); andy.hall@newcastle.ac.uk (A.G.H.); christine.harrison@newcastle.ac.uk (C.J.H.); 3 Maria Sklodowska-Curie Institute, Oncology Center, Gliwice Branch, 02-034 Warszawa, Poland; monika.pietrowska@io.gliwice.pl; 4 School of Health & Life Sciences, Teesside University, Middlesbrough TS1 3JN, UK; p.zhou@tees.ac.uk (P.Z.); v.rand@tees.ac.uk (V.R.); 5 National Horizons Centre, Teesside University, Darlington DL1 1HG, UK; 6 Department of Natural Products Biochemistry, Institute of Bioorganic Chemistry, Polish Academy of Sciences, 61-704 Poznan, Poland; lukasmar@ibch.poznan.pl; 7 Biosciences Institute, Newcastle University, Newcastle upon Tyne NE1 7RU, UK; A.Walaszczyk2@newcastle.ac.uk; 8 Newcastle University Medicine Malaysia, EduCity Iskandar, Johor 79200, Malaysia; jia.yee.ho2@gmail.com; 9 Department of Haematology, City Hospitals Sunderland NHS Trust, Sunderland SR4 7TP, UK; scott.marshall@nuth.nhs.uk

**Keywords:** chronic lymphocytic leukaemia (CLL), BACH2, BCL6, prognosticator, tumour suppressor, coimmunoprecipitation and proteomics

## Abstract

**Simple Summary:**

Chronic lymphocytic leukaemia (CLL) is the most frequent type of leukaemia with a highly variable presentation, clinical course, and outcome. The overall aim of our study was to determine the clinical and functional significance of two B-cell regulators, BACH2 and BCL6, in CLL. The results showed that CLL patients expressing low levels of *BACH2* and *BCL6* RNA had a shorter overall survival (OS). Their low RNA expression was associated with a shorter overall survival of immunoglobulin heavy chain variable region-mutated (IGHV-M) CLL patients, as well as those with 11q and 13q deletions. Further, co-immunoprecipitation and mass spectrometry studies from MEC-1 CLL cells showed no direct interaction between BACH2 and BCL6, but shared protein networks that are involved in ubiquitination mediated B-cell receptor functions, nucleic acid metabolism, protein degradation, and homeostasis in CLL biology. Taken together, this study reports BACH2 as a potential prognosticator and indicates the protein networks influenced by BCL6 and BACH2 in CLL.

**Abstract:**

Chronic lymphocytic leukaemia (CLL) is a heterogeneous disease with a highly variable clinical outcome. There are well-established CLL prognostic biomarkers that have transformed treatment and improved the understanding of CLL biology. Here, we have studied the clinical significance of two crucial B cell regulators, BACH2 (BTB and CNC homology 1, basic leucine zipper transcription factor 2) and BCL6 (B-cell CLL/lymphoma 6), in a cohort of 102 CLL patients and determined the protein interaction networks that they participate in using MEC-1 CLL cells. We observed that CLL patients expressing low levels of *BCL6* and *BACH2* RNA had significantly shorter overall survival (OS) than high *BCL6*- and *BACH2*-expressing cases. Notably, their low expression specifically decreased the OS of immunoglobulin heavy chain variable region-mutated (IGHV-M) CLL patients, as well as those with 11q and 13q deletions. Similar to the RNA data, a low BACH2 protein expression was associated with a significantly shorter OS than a high expression. There was no direct interaction observed between BACH2 and BCL6 in MEC-1 CLL cells, but they shared protein networks that included fifty different proteins. Interestingly, a prognostic index (PI) model that we generated, using integrative risk score values of *BACH2* RNA expression, age, and 17p deletion status, predicted patient outcomes in our cohort. Taken together, these data have shown for the first time a possible prognostic role for *BACH2* in CLL and have revealed protein interaction networks shared by BCL6 and BACH2, indicating a significant role for BACH2 and BCL6 in key cellular processes, including ubiquitination mediated B-cell receptor functions, nucleic acid metabolism, protein degradation, and homeostasis in CLL biology.

## 1. Introduction

Chronic lymphocytic leukaemia (CLL) is the most prevalent leukaemia among elderly people in Western countries, showing highly variable outcomes [1]. In contrast to other mature B-cell malignancies, the immunoglobulin heavy chain variable region (IGHV) genes in CLL are either unmutated or somatically mutated and are related to a poor or favourable outcome, respectively [2,3]. Although the cellular origin of CLL remains unclear, unmutated CLL (U-CLL) is likely to arise from pre-germinal centre (GC) CD5^+^ CD27^−^ B-cells [4]. These cells originate from naive B-cells or a separate lineage of precursor B-cells that have not encountered antigen to form a GC [4]. In contrast, mutated CLL (M-CLL) appears to originate from post-germinal centre CD5^+^ CD27^+^ B-cells, which are transcriptionally similar to memory B-cells that have undergone the GC reaction [5,6]. Despite antigen engagement and intact B-cell-receptor (BCR) signalling, CLL cells fail to undergo terminal differentiation.

The transcriptional repressor, BACH2, is required for the somatic hypermutation (SHM) and class switch recombination (CSR) of antibody genes, GC formation, and the inhibition of plasma cell (PC) differentiation in B-cells [7,8,9]. BACH2, in co-operation with BCL6, regulates GC B-cell fate through transcriptional and other biochemical mechanisms [10,11]. Double heterozygous *Bcl6*^−/+^ *Bach2*^−/+^ mice exhibit a profound reduction in GC formation in response to T-cell dependent antigen immunization [10]. In GC B-cells, both BACH2 and BCL6 proteins are upregulated, with the stability of the BACH2 protein regulated by BCL6 [10]. Interaction between BACH2 and BCL6 represses the transcription of PRDM1 (PR domain containing 1, with ZNF domain), a key driver of plasma cell differentiation [10]. Interestingly, the ratio of BACH2:BCL6 expression levels represent a significant predictor of outcome in acute lymphoblastic leukaemia (ALL) [12]. In diffuse large B-cell lymphoma (DLBCL), BACH2 mutations occur in about 5% [13] and BACH2 expression is a predictor of an inferior outcome in the DLBCL high-risk group [14]. Moreover, BACH2 (induced by c-Rel/NF-κB) functions as a tumour suppressor in the early stages of B-cell lymphoma development [15]. Recently, when the mRNA expression of *BACH2* was studied in untreated CLL patients with age-matched healthy donors, *BACH2* mRNA expression was shown to be reduced in CD4^+^ T-cells, CD8^+^ T-cells, and leukemic B-cells [16]. Here, we have investigated the prognostic role of BCL6 and BACH2 in a CLL patient cohort. Further, we have determined the relationship between them in MEC-1 CLL cells, using co-immunoprecipitation (Co-IP) and mass spectrometry.

## 2. Materials and Methods

This retrospective study included 102 CLL patients following informed consent. Patients were selected based on the availability of fresh samples, clinical and cytogenetic data, as well as overall survival data up to June 2015. This study cohort is heterogeneous, reflecting the diversity typically found among CLL patients. Samples were obtained through the Newcastle Biobank (17/NE/0361). All patients were diagnosed with CLL according to World Health Organization (WHO) criteria. RNA and protein extraction, qRT-PCR, Western blot, and statistical analysis from patient samples were performed using standard techniques, as detailed in the Supplementary Materials. The in vitro protein localisation and interaction studies in MEC-1 cells are also included in the Supplementary Materials.

## 3. Results

### 3.1. BACH2 and BCL6 Low RNA Expression Predicts Shorter Overall Survival in CLL

**Figure 1:**
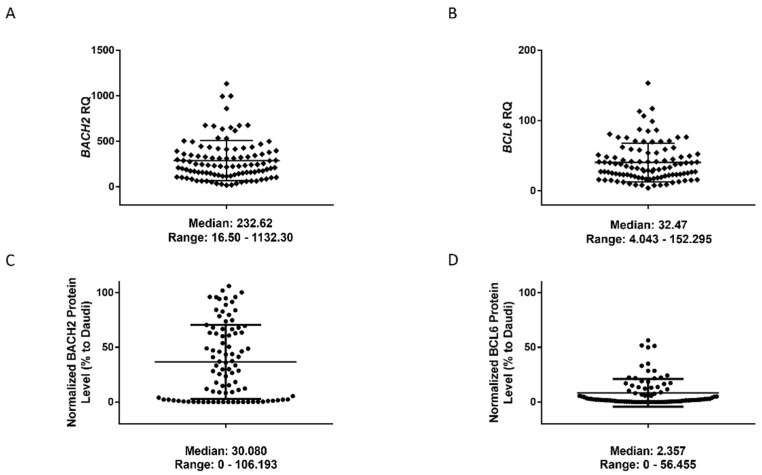
RNA and protein expression levels of BACH2 and BCL6 in CLL samples. Scatter plots showing *BACH2* RNA levels ranging from 16-fold to 1132-fold (**A**) whereas *BCL6* RNA levels range from 4-fold to 152-fold (**B**). Scatter plot showing BACH2 protein levels ranging from 0 to 106 (**C**) and BCL6 protein levels ranging from 0 to 56 (**D**).

The total RNA and protein expression of *BACH2* and *BCL6* were measured in a cohort of 102 and 91 among 102 CLL patients (Table 1 and Table S1), respectively. Expression showed a high variability between CLL samples (Figure 1, Figures S1 and S2). Receiver operating characteristic (ROC) analysis was used to assign “high” and “low” expression sets (Figure S2 and Table S2, showing the number of patients in each group of ROC analyses). A positive correlation was found between BACH2 RNA and protein levels (Figure S3A), whereas *BCL6* RNA expression did not correlate with its protein levels (Figure S3B), possibly due to the very low expression levels of BCL6 in CLL. Overall, BCL6 median expression was seven times lower than BACH2 (Figure 1). However, the correlation between RNA and protein expression data followed a similar trend, although not statistically significant (Figure S3).

**Figure 2:**
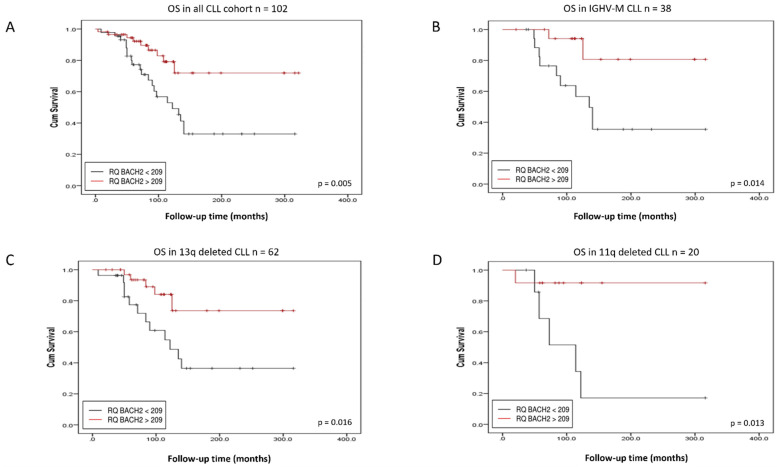
Kaplan–Meier analysis of CLL patients stratified by *BACH2* RNA expression. *BACH2* low expression predicts shorter overall survival in the whole cohort (**A**), in immunoglobulin heavy chain variable region-mutated (IGHV-M) patients (**B**), in 13q-deleted patients (**C**) and in 11q-deleted subgroups (**D**).

**Figure 3:**
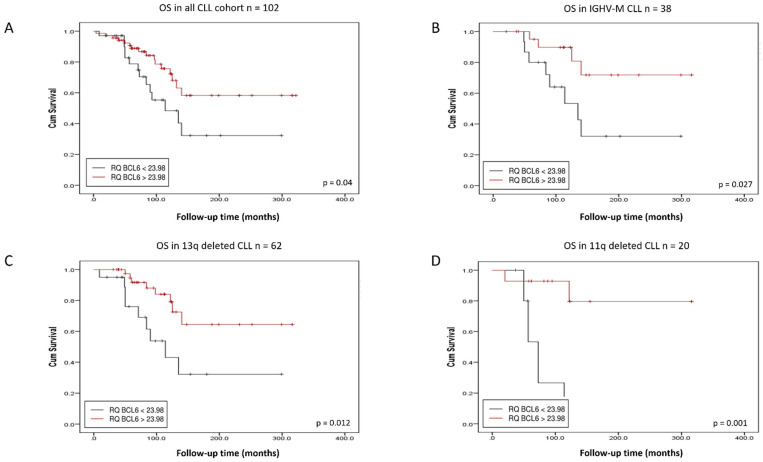
Kaplan–Meier analysis of CLL patients stratified by *BCL6* RNA expression. *BCL6* low expression predicts shorter overall survival in the whole cohort (**A**), in immunoglobulin heavy chain variable region-mutated (IGHV-M) patients (**B**), in 13q-deleted patients (**C**) and in 11q-deleted subgroups (**D**).

**Figure 4:**
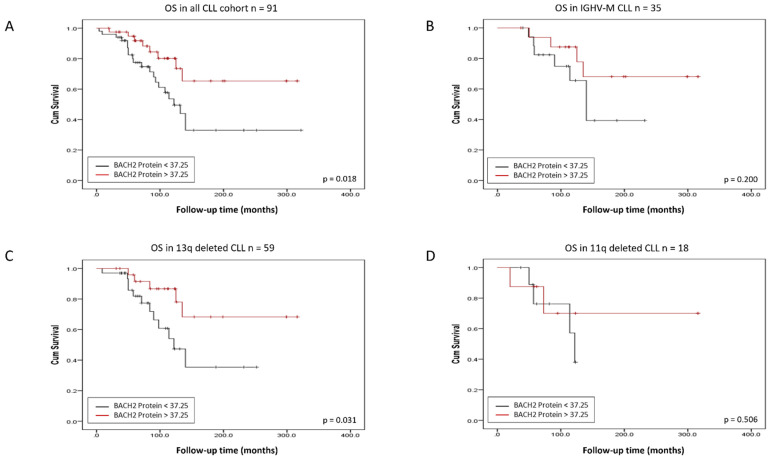
Kaplan–Meier analysis of CLL patients stratified by BACH2 protein expression. BACH2 protein low expression predicts shorter overall survival in all cohorts (**A**) and in 13q-deleted (**C**), but not in immunoglobulin heavy chain variable region-mutated (IGHV-M) patients (**B**) or 11q-deleted subgroups (**D**).

CLL expressing low levels of the *BACH2* and *BCL6* RNA had a significantly shorter overall survival (OS) than those expressing high levels (*p*-value = 0.005 and *p*-value = 0.04, respectively) (Figure 2 and Figure 3). In relation to protein expression, low BACH2 protein levels also predicted a shorter OS (Figure 4A), whereas low BCL6 protein showed only a trend towards a reduced OS (Figure S4). These findings differed from previous observations of higher expression of BCL6 correlating with a shorter treatment-free interval (TFI) in early-stage CLL [17]. While our cohort was more representative of a general CLL population (Table 1 and Table 2), it included a limited amount of data on the time to first treatment and progression-free survival of the cohort. Therefore, we limited our analyses to the overall survival in this study.

When the study cohort was separated based on treatment status (Table 2, Tables S1 and S2), the low expression of *BACH2* RNA had a significantly shorter OS than those expressing high levels in the treatment naïve cohort, indicating *BACH2* as a potential prognosticator (Figure S5). The low BCL6 protein expression based on OS analyses showed only a trend towards a reduced OS in the treatment naïve cohort but was not statistically significant. Similarly, when previously treated patients were analysed, the same trend of low levels of *BACH2* and *BCL6* expression corresponding to a shorter OS was observed (Figure S5). However, the *p*-value was not significant, possibly due to the lower number of data points. It is important to note that the treatment naïve patient numbers within the high and low expressing groups of *BACH2* and *BCL6,* separated based on the ROC analyses, included a similar number of treated and untreated patients. Among the 67 treatment naïve patients, low and high *BACH2* expressing patients included were 30 and 37, respectively. Similarly, within the CLL treated subgroup, the *BACH2* low and high expression patients included 15 and 20, respectively, demonstrating that the high and low *BACH2* expression groups were balanced.

**Figure 5:**
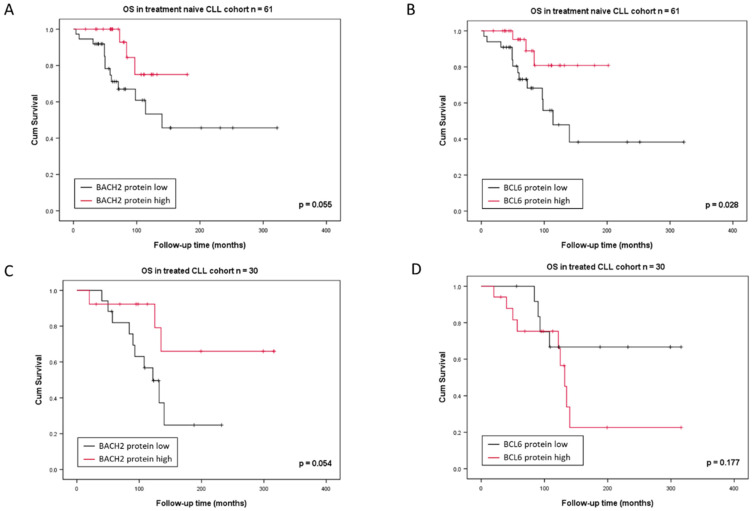
Survival analyses based on treatment status within the high and low protein expressing groups of BACH2 and BCL6: (**A**) BACH2 expression of treatment naïve arm, (**B**) BCL6 expression of treatment naïve arm, (**C**) BACH2 expression of previously treated arm, and (**D**) BCL6 expression of previously treated arm.

For survival analyses of the treatment naïve cohort, it was evident that low BACH2 as well as BCL6 protein expression predicts a shorter survival rate, with statistical significance (Figure 5A,B). Similarly, in the previously treated cohort, OS was also shorter when BACH2 protein expression was low compared to high expression, but this was not observed for BCL6 (Figure 5C,D). Therefore, the data strongly suggest BACH2 as a promising prognosticator in CLL.

### 3.2. BACH2 and BCL6 Low RNA Expression Predicts Shorter Overall Survival in IGHV-M, 13q-, and 11q-Deleted Patient Subgroups of CLL

*BACH2* and *BCL6* low RNA expression also predicted a poor outcome in IGHV-M patients (Figure 2B and Figure 3B), whereas low BACH2 protein levels showed only a trend towards a reduced OS in this subgroup (Figure 4B). Intriguingly, in the GC environment, somatic mutations in the 5′-intronic region of *BCL6* occurred in a similar manner to IGHV somatic hypermutation in CLL [18]. Although *BCL6* mutations appeared not to influence its expression, these mutations are regarded as a marker of B-cell transit through the GC, as they occur frequently in normal memory B-cells [18,19]. We found no significant correlation between expression levels of *BACH2* and *BCL6* with the common CLL prognostic factors, including clinical staging, age, and cytogenetic abnormalities (13q14, 11q23, and 17p13 deletions), but survival analysis showed that a low RNA expression of *BACH2* and *BCL6* was related to a poor outcome in the 13q- (Figure 2C and Figure 3C) and 11q-deleted subgroups (Figure 2D and Figure 3D). Low BACH2 protein levels predicted a shorter OS in 13q-deleted patients (Figure 4C), whereas BACH2 and BCL6 protein levels showed no impact on survival in 11q-deleted CLL (Figure 4D and Figure S4D). The loss of 13q14.3 is the most common chromosomal aberration in CLL, accounting for 40–60% and, as the sole abnormality, it is reported to be a good prognostic indicator [20]. In this subgroup, further reduced survival was associated with low levels of *BACH2* RNA, as well as protein (Figure 2C and Figure 4C).

### 3.3. BACH2 Is a Promising Independent and Integrative Predictor of Outcome for CLL

**Figure 6:**
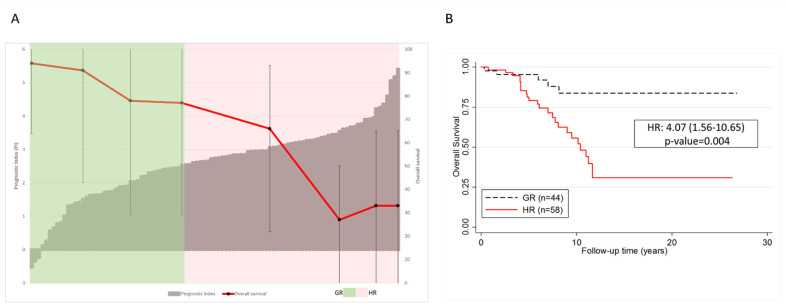
Prognostic index and overall survival (OS) rates (at 10-year intervals) of patients based on the integrative risk score values. The independent prognostic variables associated with OS were confirmed by multivariate analysis using the Cox proportional hazards model. (**A**) prognostic index model was established based on independent variables that were significantly associated with OS in the multivariate analysis (Table S3C). The PI of 2.60 was established as the optimal cut-point for this analysis. (**B**) Overall, the hazard ratio of comparing cases with PI ≥ 2.60 to cases with PI < 2.60 is 4.074 (1.568–10.655) with a *p*-value of 0.004. GR and HR are good and high risk, respectively.

The multivariate analysis confirmed the findings from the univariate analysis (Table S3), revealing that the prognostic value of *BACH2* RNA expression, in terms of OS, was independent of age and 17p deletion status—two of the most reliable prognostic factors in CLL (Table S3C). The IGHV mutational status was highly significant in both the univariate and multivariate analyses. Further, when the multivariate analysis including *BACH2* and *BCL6* RNA expression, age, 17p status, treatment status, BACH2, and BCL6 protein expression was performed, the data verified the independent prognostic value of low BACH2 expression (Tables S4 and S5). However, *BACH2* expression was not a strong prognosticator when all covariates were considered within the same model (Table S4). Moreover, a prognostic index (PI) model (Figure 6A) generated using integrative risk score values of *BACH2* RNA expression, age, and 17p deletion status predicted outcome in our cohort (Figure 6). Overall, the hazard ratio of comparing low risk cases with PI ≥ 2.60 to high-risk cases with PI < 2.60 was 4.074 (1.568–10.655), with a *p*-value of 0.004 (Figure 6B). This observation suggested that BACH2 could be a promising independent and integrative predictor of outcome for CLL, although further validation in a larger cohort is essential to confirm BACH2 as a clinically significant prognosticator in CLL.

Additionally, we found that *BACH2* RNA expression was negatively correlated with CD38 expression (Pearson’s r = −0.418, *p*-value = 0.011) (Table S3A), which is a marker for an unfavourable prognosis in CLL, which correlates with the BCR signalling response, activation, and proliferation [21]. BCL6 inhibits the expression of p53 and regulates the DNA damage-induced apoptotic responses in GC B-cells [22,23,24]; thus, we also studied the link between TP53 mutations and RNA expressions of *BCL6* and *BACH2*, which showed no correlation, implying TP53-independent functions for these regulators in CLL (Table S3A,B).

### 3.4. Mapping of BACH2 and BCL6 Mediated Signalling

**Figure 7:**
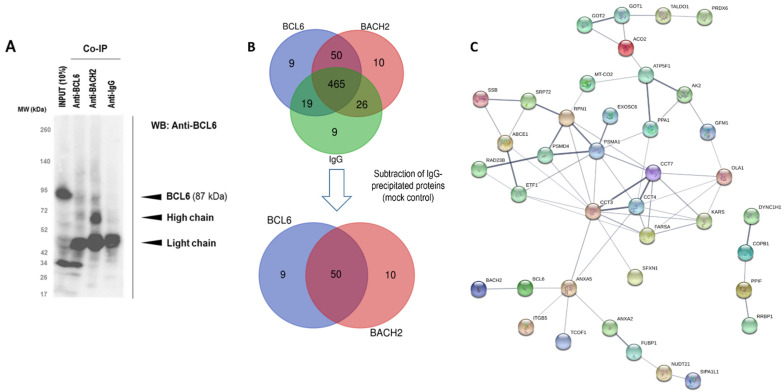
BACH2/BCL6 interactions, proteins that exclusively associate with BACH2 and BCL6 detected from the co-immunoprecipitation and subsequent MS studies. (**A**) Immunoprobing of BCL6 presence in co-immunoprecipitation (Co-IP) obtained with application of anti-BCL6, anti-BACH2, and anti-IgG (control) in MEC-1 cells (uncropped WB original image see Figure S9), (**B**) identification of proteins interacting with BCL6 or BACH2 with mass spectrometry (MS)—Orbitrap analyses, and (**C**) protein–protein interaction networks of BACH2 and BCL6.

We identified a positive correlation between BCL6 and BACH2 expression (Figure S6), suggesting a possible concerted action in CLL. This finding was in agreement with the cooperative function previously reported between BCL6 and BACH2 in GC B-cells [10]. Furthermore, we investigated BCL6/BACH2 subcellular localisation, using immunofluorescence and molecular interaction through reciprocal co-immunoprecipitation (co-IP) followed by Orbitrap mass spectrometry analyses in MEC-1 CLL cells. Immunofluorescence analyses of BACH2 (green) and BCL6 (red) showed that BACH2 was predominantly localised within the cytoplasm, whereas BCL6 was found in both the cytoplasm and nucleus in MEC-1 CLL cells (Figure S7). The co-immunoprecipitation data showed no co-IP of BCL6 and BACH2 proteins (Figure 7A), while further proteomic analyses by mass spectrometry confirmed a lack of direct interaction between them under the analysed conditions. Nevertheless, 50 proteins co-precipitated with BACH2 and BCL6, showing an extensively shared protein network between them (Figure 7 and Table S6). The number of proteins that isolated exclusively with BACH2 and BCL6 were 10 and 9, respectively (Table S7).

Among the 50 identified interaction partners, 28 proteins were localised in the cytoplasm, while 7 were in the cell periphery/membrane (DYNC1H1, COPB1, ARHGD1B, SFXN1, RRBP1, ITGB5, and RPN1). A further 15 of the interactors display catalytic activity, of which 13 are enzymes that catalyse metabolic interconversion (GANAB, NASA, PPA1, AK2, TALDO1, PRDX6, GOT1, GOT2, ECH1, MT-CO2, PLCG2, ACO2, RPN1) (Figure S8 and Table S8).

Several components of the eukaryotic 26S proteasome complex that are part of the two subcomplexes—the 20S core particle (CP) and the 19S regulatory particle [25]—are identified as interaction partners of BACH2 and BCL6. BACH2-specific and shared interaction partners include PSMD1, PSMD14, KIAA0368, PSMD4, PSMA1, proteasome adapter and scaffold proteins (ECPAS), RAD23B, ABCE1, and USP48. The ubiquitin-mediated protein degradation pathway is one of the major regulatory processes in CLL that regulates B-cell receptor functions, vesicle transport, and intracellular trafficking [26,27,28]. In addition, CLL lymphocytes are hypersensitive to apoptotic death activation through the specific inhibition of proteasome [29,30]. Conversely, the stability of BCL6 is also reported to be regulated by ubiquitylation and proteasomal degradation in DLBCL [31]. Hence, there is a possibility that BACH2 and BCL6 may be substrate adaptors for E3 ubiquitin ligases and involved in proteosome mediated degradation processes in CLL.

The second largest group of proteins that interact with BACH2 and BCL6 belong to nucleic acid metabolism (BCL6: MCM3, MCM4, BCL6 and BACH2: SSB, EXOSC6, FUBP1, TCOF1, NUDT21, RAD23B). Mini-chromosome maintenance proteins (MCMs) are a family of six structurally related helicases (MCM2–7) that play critical roles in DNA replication and genome stability by forming a variety of complexes [32,33]. Each subunit of MCM has distinct functions during the initiation, elongation, and termination processes of replication, as well as in maintaining genomic stability [34]. Consequently, they have been implicated in several cancers, including DLBCL [35]. In CLL, increased expression of MCM2, MCM3, and MCM7 have been observed in primary samples from patients and two CLL-derived cell lines (the MEC-1 and EHEB cell lines) [36]. Further, MCM7 suppression amplifies replication stress and genomic instability and, in turn, hypersensitizes cells to certain drugs such as fludarabine, which is used as a first-line therapy for CLL [36]. Thus, existing reports and our findings indicate that the association between BCL6 and MCM components influence novel regulatory mechanisms in DNA damage response and genome stability. In the context of BACH2 in DNA damage, recent microarray studies have reported a strong downregulation of BACH2 upon UV-induced damage or aging [37]. In addition to the above identified major interaction networks, the interaction partner RAD23B, found in our study, connects DNA repair and proteasome pathways [38]. The carboxy terminus of RAD23B binds to the RAD4 DNA repair protein and the N terminal ubiquitin-like domain interacts with the 26S proteasome and coordinates the regulation of these processes [39].

In the same theme of ubiquitination, genome stability, and DNA damage, TCOF1 (treacle ribosome biogenesis factor 1) has also been reported to function in ubiquitination through E3 ubiquitin ligase complex, RNA biogenesis, mitosis, proliferation, DNA damage response, and apoptosis [40,41]. There are reports that connect FUBP1 (far upstream element binding protein 1) and NUDT21 to the RNA binding, polyadenylation, and fine tuning of protein and RNA levels [41,42]. They both have been identified as potent pro-proliferative and anti-apoptotic factors by the modulation of complex networks in hematologic disorders and solid tumours [40,41]. Similarly, the BACH2–BCL6 interactors, EXOCS2 and SSB, also reported to interact with RNA and EXOCS2, are part of the highly conserved RNA processing/degrading exosome complex proteins in erythroid differentiation [43,44]. Further, in the process of erythroid maturation blockade, GATA-1 and Foxo3 transcriptionally regulate exosome complex components and BACH2 has been reported to function with both in T cell differentiation and homeostasis [45]. Thus, this collection of interaction partners indicates cross communications mediated by BCL6 and BACH2 in key genome stability, the ubiquitin-mediated protein degradation pathway, and homeostasis processes in CLL.

## 4. Discussion

The precise cellular origin of CLL remains unclear and the current knowledge of CLL biology demonstrates no direct link between the proliferative circulating CLL cells to a specific normal B-cell subset [46,47]. B-cell associated gene-signatures are separated based on pre- and post-GC B-lymphocytes (pre GC: pre-BI, pre-BII, and immature; post GC: naïve, memory, or plasma cell subtypes) [47,48]. Several lines of evidence have suggested that BCL6 and BACH2 cooperate in GC B-cells [49,50,51], thus understanding the clinical significance of such transcriptional regulators in CLL patients and the protein networks influenced by them is of high importance in CLL treatment. The expression of BCL6 and the synergistic gene repression function of BCL6 and BACH2 are essential for the highly complex GC formation process, which is orchestrated by key molecular regulators such as MEF2B, IRF8, IRF4, BLIMP1, and *TP53* [23,52]. Here, in this retrospective study, we have investigated the clinical significance of BACH2 and BCL6 in a typical, heterogenous CLL patient cohort that includes treatment naïve and treated subgroups. We have shown that low RNA expression of *BACH2* and *BCL6* predicts a shorter OS in IGHV-M, 13q-deleted (regarded as a known low-risk subgroup), and 11q-deleted subgroups. In addition, low BACH2 RNA and protein expression also predicted a shorter OS in treatment naïve CLL patients. The number of treated and treatment naïve patients were fairly equally distributed and balanced within most of the subgroups used in the univariate and multivariate analyses (Table 1, Tables S1 and S2). However, when the number of patients within the different molecular subgroups, such as IGHV-M, 13q-, and 11q-deleted, were further divided based on treatment status, the number of patients in each subgroup (e.g., 13q-deleted and 11q-deleted, high or low BACH2 expressing treatment naïve or treated group, Table S2) were too small to perform meaningful analyses. Therefore, the BACH2 prognosticator role within these subgroups may be confounded by the treatment status of the patients. However, the clear trend observed in this overall survival study suggests a promising tumour suppressor function for BACH2 and BCL6. Although the relationship between median progression-free survival (PFS)/time-to-progression (TTP) and median overall survival in CLL appears to strongly correlate (in second- and subsequent-line therapies, but not in the first-line setting), and PFS and TTP are suggested as possible surrogates of overall survival [53], it was not possible to perform the progression-free survival and time to first treatment analyses using this CLL study cohort due to the data limitations.

The deletion of 13q14 is the most frequent genetic lesion in CLL, being most prevalent in IGHV-M CLL [54,55]. It is noteworthy that the minimal region of 13q-deletion includes micro RNAs, MIR15A–MIR161, that regulate cell cycle and apoptosis in B-cells [56,57,58,59]. Similarly, during the course of the B-cell development, BACH2 has also been reported to play significant cell cycle and apoptotic regulatory roles [59,60,61], suggesting a possible tumour suppressor function for BACH2 in good risk subgroups of CLL. In contrast, in DLBCL, BACH2 expression has been reported as a predictor of poor prognosis, especially in the high-risk group [14]. Although no direct experimental evidence on the mechanism that identifies BACH2 as a poor prognosticator in DLBCL is available, these observations are opposite to the those reported in this study. It is plausible that the expression of the BACH2 regulator, PRDM1, or BLIMP1, which controls plasma cell differentiation, is required for the terminal differentiation of B-cells, which is strongly suppressed by BACH2 in normal B-cell development. In DLBCL, the expression of PRDM1 appears to be very weak in lymphoma cells, hence the constitutive expression of BACH2 may contribute to maturation arrest of lymphoma cells, leading to lymphomagenesis. In CLL, a recent study showed a decrease of BACH2 and an increase of PRDM1 in B-cells from CLL patients [52], suggesting an opposite effect to DLBCL. However, further experiments and investigations focused on additional clinical samples are essential to understand the seemingly distinct BACH2–PRDM1 mediated regulatory mechanisms in DLBCL and CLL.

The prognostic role of *BCL6* RNA and protein expression, as well as its mutational status in CLL, have been investigated over the years with conflicting reports [54,62,63], possibly resulting from differences in cell types tested and variations in the technologies deployed [17,63,64,65]. The lack of correlation between the RNA and protein levels of BCL6 observed in our data is, however, not unusual [66,67], as the integration of transcriptomic and proteomic data from normal as well as various cancer tissues have highlighted the non-linear relationship between RNA and protein levels (i.e., ~60% of the variation between RNA and protein abundances) [68,69]. This observation may result from the complex, poorly understood intracellular transcriptional or post-transcriptional processes that regulate the rates of RNA and protein production/turnover/stability and their mode of regulation under different cellular conditions [66,67,68,69].

Our co-immunoprecipitation studies, performed in MEC1 CLL cells, show no direct interaction between BACH2 and BCL6, but the majority of the interaction partners found in the shared protein interaction network suggested cooperation between them in key signalling processes, such as the ubiquitin-mediated protein degradation pathway, nucleic acid metabolism, and cellular homeostasis (Table S7). Further, the interaction partners of BACH2 and BCL6 appeared to participate in cytoskeletal signalling, vesicle transport molecules (BACH2: RHOG and DSP BCL6: ARF3, STXBP2, PHB2, and PSAP) and spliceosome machinery elements (BACH2: RETSAT, RPS14, SART3, BCL6: CTNNBL1). The role of cytoskeletal signalling, vesicle transport, spliceosome machinery, and the ubiquitin proteasome system in haematological malignancies, including CLL, has also been suggested previously [70,71,72,73]. Further functional validation of these protein networks in primary CLL cells and model systems is essential to determine the exact roles of BACH2 and BCL6 in CLL.

## 5. Conclusions

Taken together, our data for the first time demonstrate *BACH2* as a possible independent, integrative predictor of outcome in CLL. The clinical outcome and the proteomic data highlight the tumour suppressor role and the functional significance of BACH2 and BCL6 in CLL biology. Further investigations that study the impact of the BACH2 and BCL6 signalling axis will not only improve our understanding of CLL pathogenesis, but will also offer new opportunities for novel targeted therapy development.

## Figures and Tables

**Table 1:** Clinical and molecular characteristics of the CLL cohort.

Characteristics, *n* = 102 Unless Stated	*N*	%
Age ≥ 65	31	62
Male	71	69.6
TP53 gene status *n* = 100		
M	12	12
UM	88	88
ZAP70 status *n* = 19		
>20%	9	47.4
<20%	10	52.6
CD38 status *n* = 36		
>20%	13	36.1
<20%	23	63.9
Treatment status *n* = 102		
Treated	35	34.3
Untreated	67	65.7

**Table 2:** Clinical and molecular characteristics of the CLL cohort including treatment status.

Binet Stage *n* = 90	*N*	%	Treated	%	Untreated	%
A	49	54.4	10	20.4	39	79.6
B	16	17.8	7	43.8	9	56.3
C	25	27.8	16	64.0	9	36.0
**IGHV status *n* = 63**			**Treated**		**Untreated**	
M	38	60.3	17	44.7	21	55.3
UM	25	39.7	7	28.0	18	72.0
**Cytogenetics *n* = 101**			**Treated**		**Untreated**	
13q	62	61.4	21	33.9	41	66.1
11q	20	19.8	11	55.0	9	45.0
12+	6	5.9	4	66.7	2	33.3
17p	8	7.9	1	12.5	7	87.5
Normal karyotype	24	23.8	18	75.0	6	25.0
**Cytogenetics *n* = 101**			**BACH2 High**		**BCL6 High**	
13q	62	61.4	35	56.5	42	67.7
11q	20	19.8	12	60.0	14	70.0
12+	6	5.9	2	33.3	4	66.7
17p	8	7.9	3	37.5	4	50.0
Normal karyotype	24	23.8	12	50.0	15	62.5

M—mutated; UM—unmutated.

## Data Availability

The data presented in this study are available in the Supplementary Materials.

## Supplementary Materials

The following are available online at www.mdpi.com/article/10.3390/cancers14010023/s1. The Supplementary material includes detailed description of all the materials and methodologies used in this study and the following figures and tables. Figure S1: (A) Example of Western blot showing BACH2 and BCL6 protein expression in 9 CLL protein lysates. Burkitt’s lymphoma cell lines Raji and Daudi were used as negative and positive controls for BACH2, respectively. (B) Antibodies’ names, supplier, and dilution factor used for Western blotting, Figure S2: Receiver operating characteristic (ROC) curves to identify the optimal cut off for BACH2 and BCL6 RNA and protein levels. ROC analysis identified 209-fold as the best *BACH2* RNA expression cut off for predicting overall survival in our cohort (A) and 23.98-fold as the best *BCL6* RNA expression cut off for predicting overall survival in our cohort (B). ROC analysis identified 37.25 as the best BACH2 protein expression cut off for predicting overall survival in our cohort (C) and 4.734 as the best BCL6 protein expression cut off for predicting overall survival in our cohort (D), Figure S3: Correlation analysis for BACH2 and BCL6 expression levels. A positive correlation was found between *BACH2* RNA (A) and protein levels whereas no correlation was found between *BCL6* RNA and protein level (B), Figure S4: Kaplan–Meier analysis of CLL patients stratified by BCL6 protein expression. BCL6 protein low expression shows a trend in predicting shorter overall survival in all cohorts (A). BACH2 protein expression does not predict outcome in immunoglobulin heavy chain variable region-mutated (IGHV-M) patients (B), 13q-deleted (C), or 11q-deleted subgroups (D), Figure S5: Survival analyses based on treatment status within the high and low expressing groups of BACH2 and BCL6, (A) BACH2 expression of treatment naïve arm, (B) BCL6 expression of treatment naïve arm, (C) BACH2 expression of previously treated arm, (D) BCL6 expression of previously treated arm, Figure S6: A positive correlation was found between RNA (A) and protein (B) levels of BACH2 and BCL6, indicating the synergistic action of both molecules in CLL, Figure S7: (A) Immunofluorescence analyses of BACH2 (green) and BCL6 (red) cellular localisation. (B) Detection of BCL6 and BACH2 localisation (cytoplasmic, nuclear, or cytoplasmic and nuclear) with application of anti-BCL6 and anti-BACH2 in MEC-1 cells. (C) Antibodies used in immunofluorescence analysis, Figure S8: Gene ontology (GO) term analyses of proteins interacting with both BACH2 and BCL6. Graphs represent number of genes observed in biological process (A) and cellular component (B) pathway analyses, (C) represents the protein–protein interaction networks of BACH2 and BCL6, Figure S9: Uncropped WB original images, Table S1: Treatment regime of the thirty-five treated patients in this study, Table S2: (A) The number of patients who had “high” and “low” BCL6 and BACH2 expression, (B) the number of patients in each molecular group separated as “high” and “low” expression levels based on ROC analyses, Table S3: (A) Correlation analysis between BACH2 RQ and the most common CLL prognosticators. (B) Correlation analysis between BCL6 RQ and the most common CLL prognosticators. (C) Univariate and multivariate analysis for overall survival, Table S4: Univariate (A) and multivariate (B and C) analyses when all the covariates are considered in the same model, Table S5: Multivariate analyses when all the covariates are considered in the same model for treatment status and protein expression levels of BACH2 and BCL6, Table S6: Proteins co-immunoprecipitated with BACH2 or BCL6 detected from the co-immunoprecipitation and subsequent MS studies, Table S7: Proteins and their function that exclusively associate with BACH2 and BCL6 detected from the co-immunoprecipitation and subsequent MS studies, Table S8: Gene ontology (GO) term analyses of proteins interacting with both BACH2 and BCL6 in biological processes and cellular components.
