# Supplementary material for: Low BACH2 Expression Predicts Adverse Outcome in Chronic Lymphocytic Leukaemia
Source: Cancers (Basel). 2021 Dec 21;14(1):23. doi: 10.3390/cancers14010023 (PMC8750551; doi:10.3390/cancers14010023)
Supplement: Supplementary file 1 [file cancers-14-00023-s001.zip › cancers-1459267-supplementary/cancers-1459267-supplementary-final.pdf]

cancers

MDPI

Supplementary Materials

Low BACH2 Expression Predicts Adverse Outcome in Chronic Lymphocytic Leukaemia

Carmela Ciardullo, Katarzyna Szoltysek, Peixun Zhou, Monika Pietrowska, Lukasz Marczak, Elaine Willmore, Amir Enshaei, Anna Walaszczyk, Jia Yee Ho, Vikki Rand, Scott Marshall, Andrew G. Hall, Christine J. Harrison, Meera Soundararajan and Jeyanthy Eswaran

1. Methods

1.1. RNA extraction and qPCR

Total RNA was extracted from peripheral blood mononuclear cells (PBMCs) using GeneJet RNA purification kit (Thermo Scientific). Concentration and purity of the total RNA samples were measured using the NanoDrop ND-1000 Spectrophotometer. cDNA was synthesized with High-Capacity cDNA synthesis kit (Life Technologies) following manufacturer's protocol. Relative quantification of *BCL6* and *BACH2* RNA expression was performed by qPCR based on TaqMan chemistry and carried out in an Applied Biosystems ViiA 7 Real Time PCR System (Applied Biosystems). *GAPDH* was used as reference for the normalization of qPCR and a pool of 5 RNA extracted from peripheral blood of 5 healthy donors was used as a calibrator. All qPCR reactions were performed in duplicate to assess data reproducibility.

TaqMan probes used for this study were: Hs02758991_g1 (*GAPDH*), Hs00222364_m1 (*BACH2*), Hs00153368_m1 (*BCL6*).

1.2. RNA expression and Data Analysis

Calculations for RNA expression were made using the comparative CT ($2^{-\Delta\Delta Ct}$) method. The potential diagnostic value of BCL6 and BACH2 RNA and protein expression was assessed by a receiver operating characteristic (ROC) curve to identify optimal cut-off point (Figure S2).

BCL6 and BACH2 expression was then split at this diagnostic cut-off and the resulting dichotomous variable was included in a Kaplan-Meier overall survival (OS) analysis (Figure 2,3). Prognostic variables associated with OS were identified through univariate analysis by Cox regression models. The hazard ratio (HR) and 95% confidence interval (CI) were calculated using Cox regression models. The independent prognostic variables associated with OS were confirmed by multivariate analysis using Cox proportional hazards model. A prognostic index model was established based on independent variables that were significantly associated with OS in the multivariate analysis (Figure 5). Any differences between OS curves were evaluated using the log-rank test. In *BACH2* and CD38 expression correlation analysis, *BACH2* and CD38 expression were treated as categorical variables, considering *BACH2* expression > 209 as 'high' and BACH2 expression < 209 as 'low', CD38 > 20% as 'high' and CD38 < 20% as low. All statistical calculations were performed using GraphPad Prism 6, STAT 14.1 and SPSS Statistics v23. Pearson's test was used to assess the correlation between variables. Differences were considered statistically significant when p value was < 0.05.

1.3. Protein level estimation using western blot

The protein expression of BACH2 and BCL6 was confirmed using the following western blot protocol. Whole cell protein lysates were extracted from CLL cells by Pierce™ RIPA buffer (Thermo Scientific) supplemented with Halt™ Protease and Phosphatase Inhibitor Single-Use Cocktail (Thermo Scientific). Protein was then quantified by Pierce™

BCA Protein Assay Kit (Thermo Scientific). Equal amount of protein from each sample were loaded in 8% SDS-polyacrylamide gel electrophoresis, transferred to polyvinylidene difluoride (PVDF) membrane (Millipore) and blocked with 5% skimmed milk in Tris-Buffered Saline (TBS) with 0.1% TWEEN 20 (Sigma-Aldrich). Then PVDF membrane was incubated overnight at 4°C with primary antibody (Figure S1B) in 1% skimmed milk in TBS with 0.1% TWEEN 20, followed by corresponding secondary antibody for 1 hour at room temperature. PVDF membrane was then developed with Amersham ECL Western Blotting detection reagents (GE Healthcare) and detected by Amersham Hyperfilm ECL (GE Healthcare). To reprobe with a different antibody, PVDF membrane was stripped with ReBlot Plus Strong Antibody Stripping Solution (Millipore). Protein bands were semi-quantified by densitometry with ImageJ software. β-Actin was used as an endogenous control for normalisation of protein expression, and Daudi protein lysate was used as a calibrator for percentage of relative protein levels. All antibodies used in this study are listed in Figure S1B.

1.4. Prognostic index model generation

Prognostic variables associated with OS were identified through univariate analysis by Cox regression models. The hazard ratio (HR) and 95 % confidence interval (CI) were calculated using Cox regression models. A two-tailed p value of <0.05 was considered statistically significant. The independent prognostic variables associated with OS were confirmed by multivariate analysis using Cox proportional hazards model using forward selection. The significance of adding new variable to the model was measured using likelihood-ratio test. A prognostic index model was established based on independent variables that were significantly associated with OS in the multivariate analysis. The prognostic index then divided into two categories based on the death rates. The independent prognostic variables associated with OS were confirmed by multivariate analysis using Cox proportional hazards model. A prognostic index model was established based on independent variables that were significantly associated with OS in the multivariate analysis (Table S2C). The PI of 2.60 was established as optimal cut-point for this analysis.

1.5. In vitro analyses

Experimental model: Human chronic B cell leukaemia MEC-1 cell line (DSMZ no: ACC 497) was used as an experimental model. Cells were grown in IMDM medium supplemented with 10% fetal bovine serum (Gibco) under standard conditions, at 37 °C in a humidified 5% CO_2 atmosphere. In some experiments, aiming to validate BACH2 and BCL6 cellular localization under stress conditions, cells were treated with proteasome inhibitor - bortezomib (Cell Signaling Technology, cat.no 2204) at a final concentration equal to 10 nM for all time points of sample collection from 4–24 hrs. Alternatively cells were also treated with corresponding concentration of DMSO (compound vehicle solution) as a control, mock sample. In order to perform proteomic studies (Western blot analyses) or immunofluorescence staining, cells were seeded in 12-well plates at a concentration equal to 2 x 10^6 or 1 x 10^6 cells/well, respectively and subjected to the bortezomib treatment. Cells were collected at a given time point and further analysed.

Immunocytofluorescence analyses: 1x10^6 cells were transferred from cell culture and washed with PBS by centrifugation. Cells were fixed in 0.4% formaldehyde/PBS for 20 minutes, permeabilized with 0.3% Triton X100/PBS and smeared on a glass microscopic slide. Cells were washed twice with PBS/0.1% Tween-20 followed by blocking with 5% BSA in PBS/0.1% Tween-20 for 1 hour. Slides were incubated overnight in humidified chambers with Anti-BACH2 or Anti-BCL6 (diluted 1:100) in blocking solution at 4°C. Slides were washed three times with PBS/0.1% Tween-20 for 5 minutes and incubated for 1 hour in humidified chambers with secondary antibody (anti-rabbit IgG) conjugated with AlexaFluor 488 followed by PBS/0.1% Tween-20 rinse. Slides were incubated with Cyto-Painter Phalloidin-iFluor 680 Reagent (Abcam) for 20 minutes in room temperature, rinsed with PBS and mounted with VECTASHIELD® Antifade Mounting Medium with

DAPI (Vectashield; DAKO). Slides were analysed with epifluorescent microscope (ZEISS Axio Imager Z1). All antibodies used are listed in Figure S1B

Co-immunoprecipitation: Each antigen sample was prepared from 50 x 10^6 cells which were transferred from the cell culture, washed with PBS by centrifugation and lysed with an ice-cold lysis buffer (25 mM Tris pH 7.4, 150 mM NaCl, 1 mM EDTA, 1% NP40, 5% glycerol and protease inhibitor cocktail) for 5 minutes. Obtained cell lysate was centrifuged at 13,000g at 4 °C for 10 minutes and collected supernatant was immediately subjected to co-immunoprecipitation procedures with application of Pierce Crosslink Magnetic IP/Co-IP Kit (Thermo Scientific, cat. no: 88805). 50 ng of Anti-BACH2, Anti-BCL6 or Anti-IgG (isotype control) antibody was coupled onto Protein A/G Magnetic Beads, covalently crosslinked with disuccinimidyl suberate (DSS) and incubated overnight at 4 °C on a rotator with an antigen sample. Beads were washed with ultrapure water to remove non-bound material and eluted in a low-pH Elution buffer. Such obtained Co-IP samples were further analyzed with Western blot and Mass spectrometry-based approaches. All antibodies used in this study are listed in Figure S1B

1.6. Mass spectrometry analyses

Sample preparation: Co-IP samples were mixed with lysis buffer (0.1 M Tris-HCL pH 8.0, 0.1 M DTT, 4% SDS) at 1:9 ratio and denatured for 10 minutes at 99°C followed by incubation at 80°C for 2h with mixing (750rpm). Next, the tubes were cooled down at 4°C for 1min and centrifuged for 15min at 14000g. The supernatant was used for protein quantification by the tryptophan fluorescence method (1) and processed using the filter-aided sample preparation (FASP) protocol as described in previous work (2). The mass spectrometry analyses were carried out as described previously (3) and the protein network and pathway influenced by the proteins detected from the co-immunoprecipitation and mass spectrometry analyses were determined using PANTHER Pathway: An Ontology-Based Pathway Database Coupled with Data Analysis Tools (4) and STRING, Protein-Protein Interaction Networks Functional Enrichment Analysis (5).

2. Figures and Tables

A

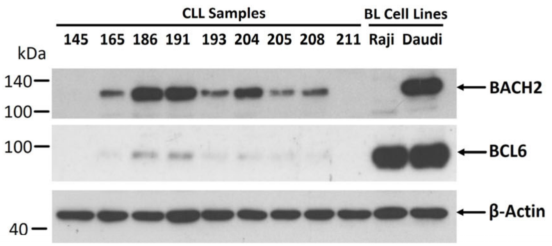

B

Antibody	Supplier	Catalogue Number	Dilution
BACH2	Cell Signaling Technology	#80775	1/1000
BACH2	Millipore	ABN171	1/1000
BCL6	Spring Bioscience	SP155	1/1000
β-Actin (HRP)	Abcam	ab49900	1/20000
Goat anti Rabbit IgG (HRP)	Dako	P0448	1/5000

Figure S1. (**A**) Example of Western blot showing BACH2 and BCL6 protein expression in 9 CLL protein lysates. Burkitt's lymphoma cell lines Raji and Daudi were used as negative and positive controls for BACH2, respectively. (**B**) Antibodies names, supplier and dilution factor used for Western blotting.

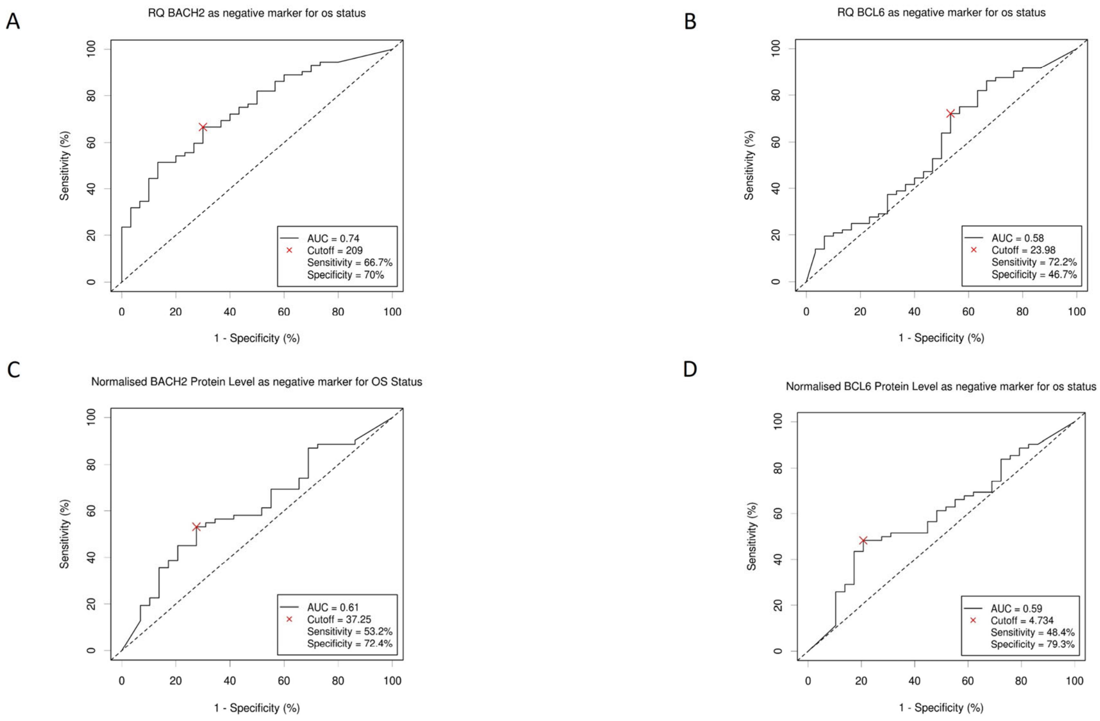

Figure S2. Receiver operating characteristic (ROC) curves to identify the optimal cut off for BACH2 and BCL6 RNA and protein levels. ROC analysis identified 209-fold as the best *BACH2* RNA expression cut off for predicting Overall Survival in our cohort (A) and 23.98-fold as the best *BCL6* RNA expression cut off for predicting Overall Survival in our cohort (B). ROC analysis identified 37.25 as the best BACH2 protein expression cut off for predicting Overall Survival in our cohort (C) and 4.734 as the best BCL6 protein expression cut off for predicting Overall Survival in our cohort (D).

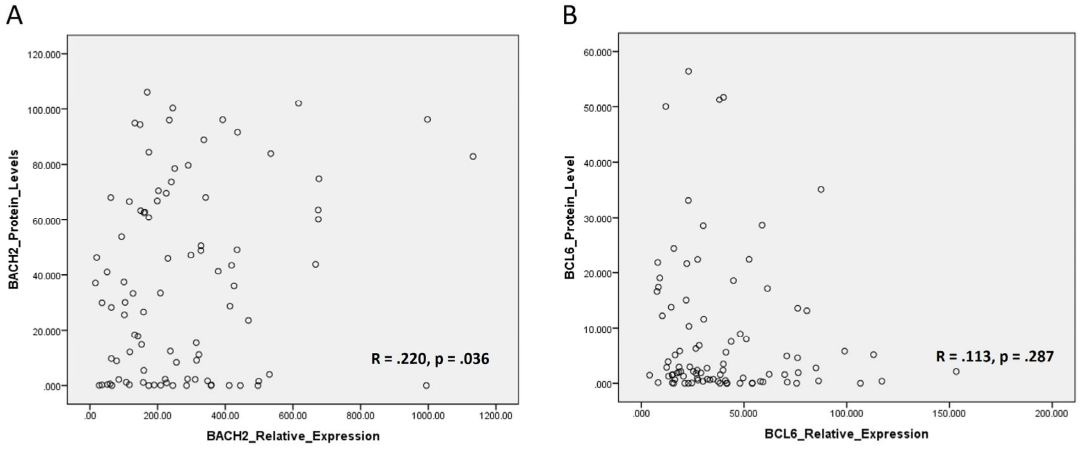

Figure S3. Correlation analysis for BACH2 and BCL6 expression levels. A positive correlation was found between BACH2 RNA (A) and protein levels whereas no correlation is found between BCL6 RNA and protein level (B).

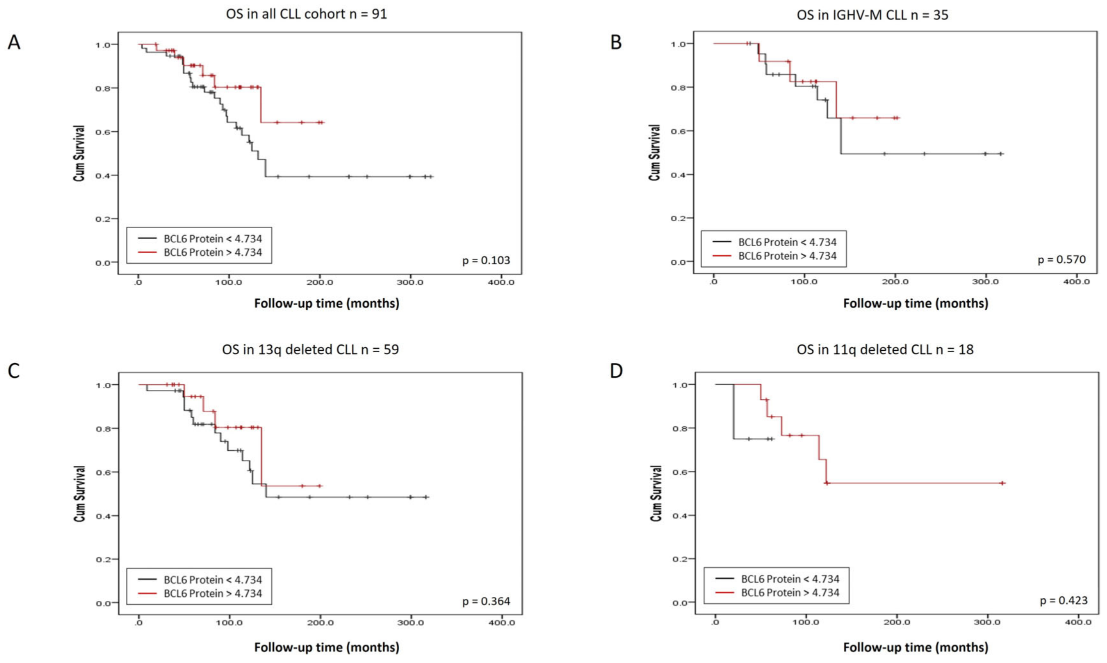

Figure S4. Kaplan-Meier analysis of CLL patients stratified by BCL6 protein expression. BCL6 protein low expression shows a trend in predicting shorter overall survival in whole cohort (A), BCL6 protein expression does not predict outcome in IGHV-mutated (B), 13q-deleted (C), or 11q-deleted subgroups (D).

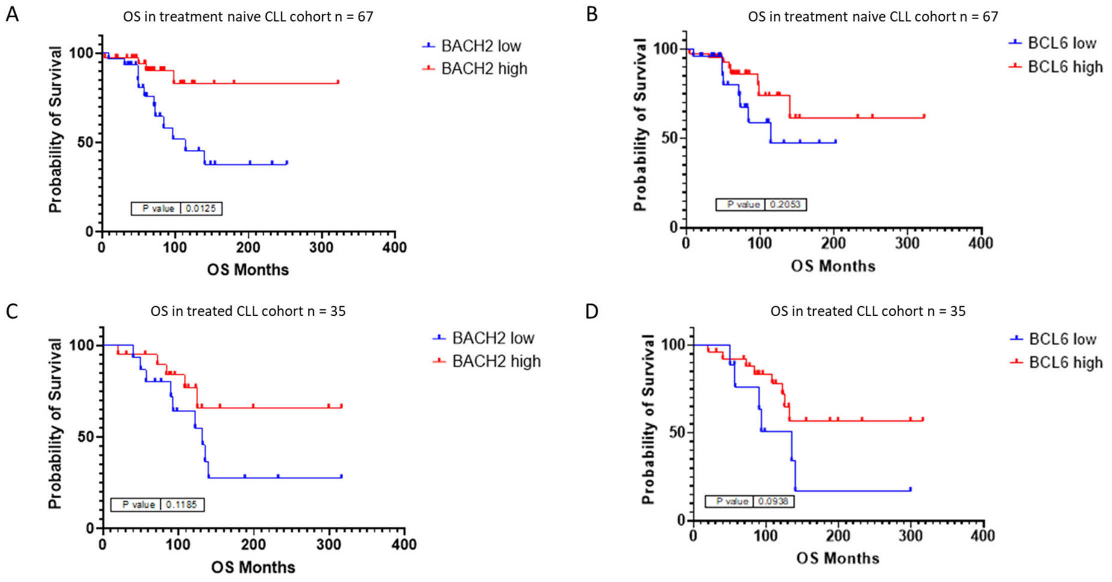

Figure S5. Kaplan–Meier analyses based on treatment status within the high and low RNA expressing groups of *BACH2* and *BCL6*, (A) *BACH2* expression of treatment naïve arm, (B) *BCL6* expression of treatment naïve arm, (C) *BACH2* expression of previously treated arm, (D) *BCL6* expression of previously treated arm.

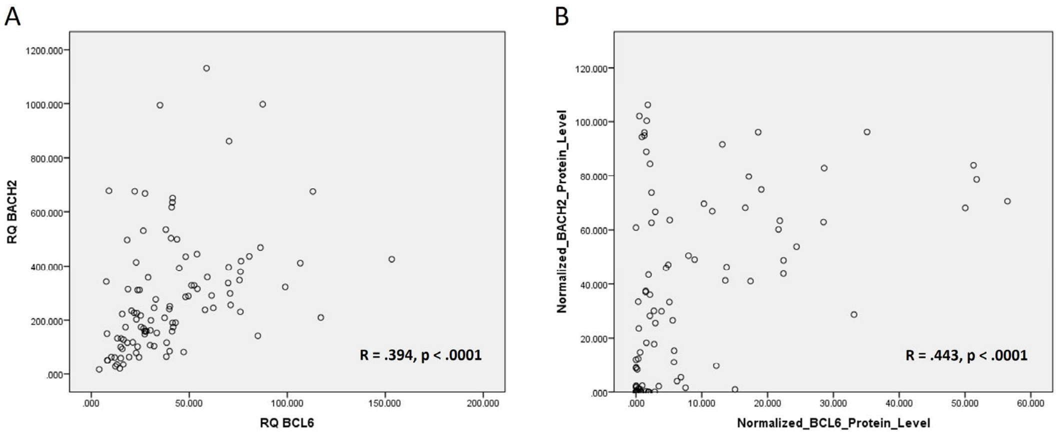

Figure S6. A positive correlation is found between RNA (A) and protein (B) levels of BACH2 and BCL6 indicating synergistic action of both molecules in CLL.

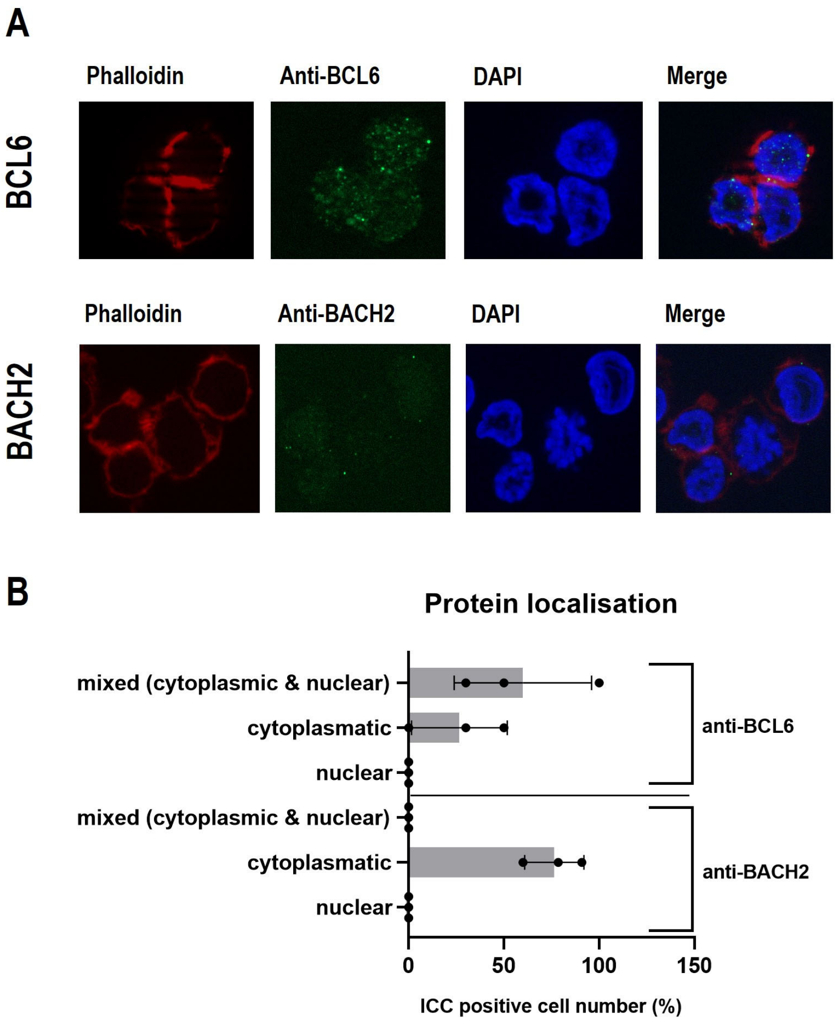

C

Antibody	Supplier	Catalogue Number
BACH2	Abcam	ab220988
BCL6	Cell Signaling Technology	#14895
Goat anti-rabbit IgG (Alexa Fluor 488)	Invitrogen	A11008

Figure S7. (A) Immunofluorescence analyses of BACH2 (green) and BCL6 (green) cellular localisation, (B) Detection of BCL6 and BACH2 localisation (cytoplasmic, nuclear or cytoplasmic & nuclear) with application of Anti-BCL6, Anti-BACH2 antibodies in MEC-1 cells, (C) Antibodies and associated supplier with its catalogue number used in immunofluorescence analysis.

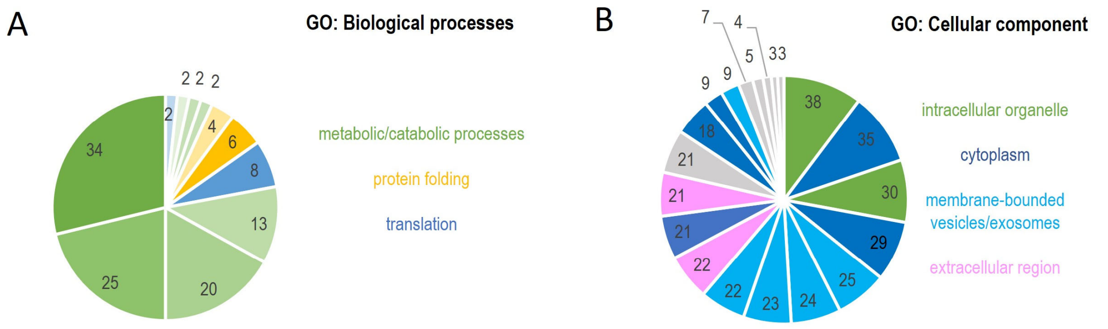

Figure S8. Gene Ontology (GO) term analyses of proteins interacting with both BACH2 and BCL6. Graphs represents number of genes observed in biological process (A) and cellular component (B) pathway analyses.

Uncropped WB original image for Main text: Figure 7—Immunoprobing of BCL6 presence in co-immunoprecipitation (Co-IP) obtained with application of anti-BCL6, anti-BACH2 and anti-IgG (control) in MEC-1 cells.

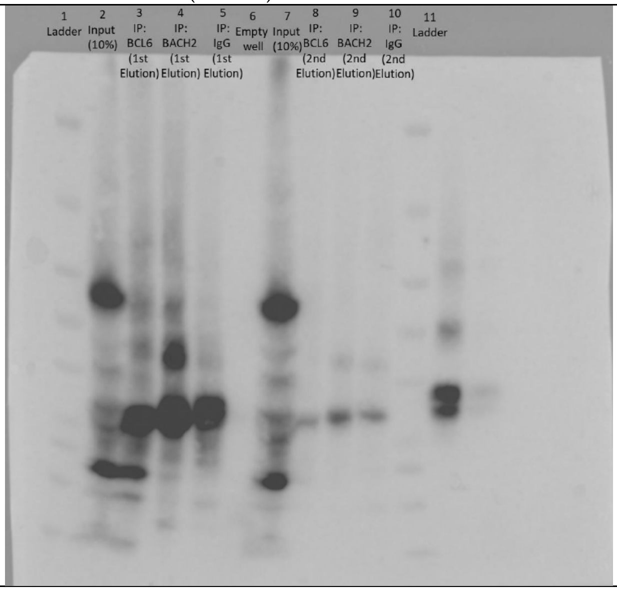

Uncropped WB original image for Supplementary material: Figure S1 (A) Example of Western blot showing BACH2 and BCL6 protein expression in 9 CLL protein lysates. Burkitt's lymphoma cell lines Raji and Daudi were used as negative and positive controls for BACH2, respectively.

BACH2

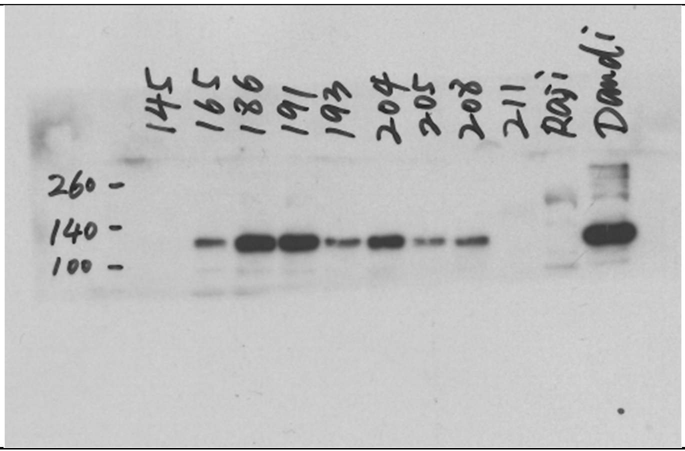

BCL6

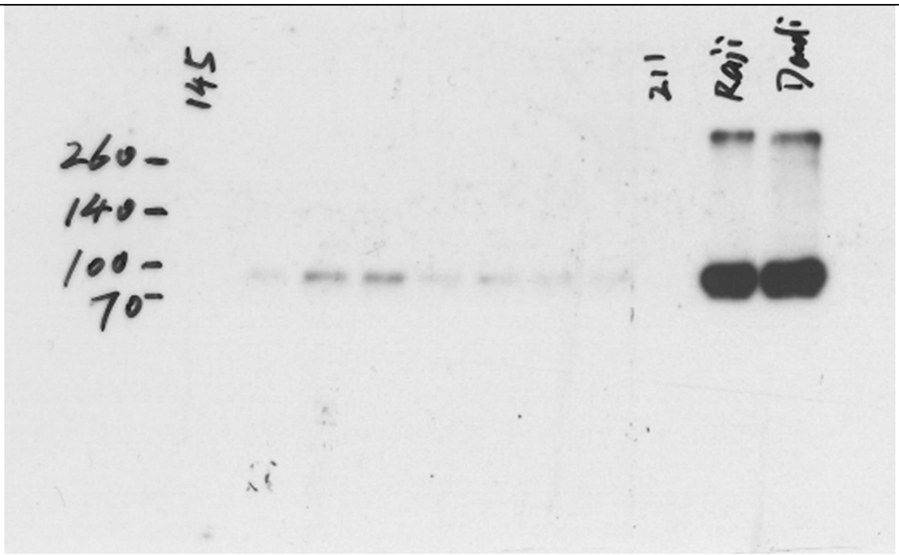

β-Actin

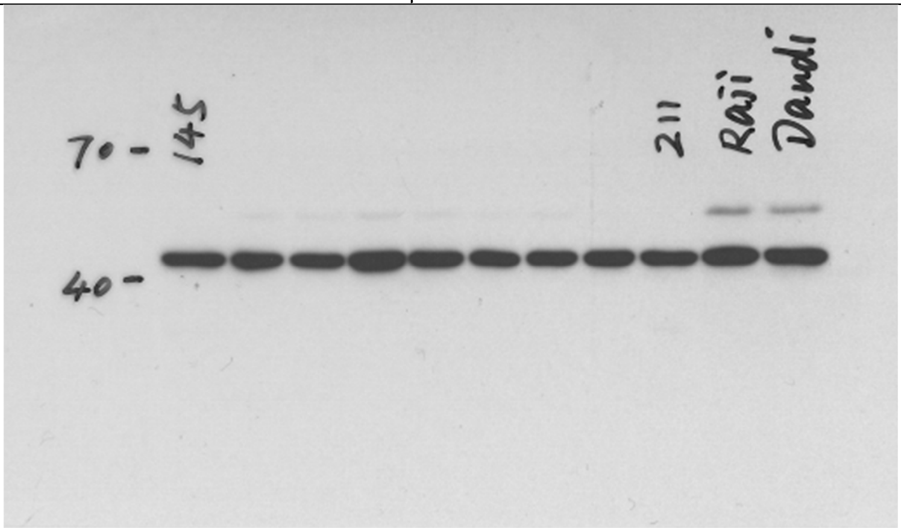

Figure S9. Uncropped WB original images.

Table S1. Treatment regime of the thirty-five treated patients in this study.

**Treatment regime, *n* = 35**	***N***	**%**
C, FC	1	2.9
CF	1	2.9
CLB alone	9	25.7
CLB, Benda	1	2.9
CLB, F	2	5.7
CLB, F, FC	1	2.9
CLB, FC	3	8.6
CLB, FC, FCR	2	5.7
CLB+pred, R-CHOP, HDMP	1	2.9
F alone	1	2.9
F, CLB, FC, FCR	1	2.9
F, FC	1	2.9
FC	2	5.7
FCC	1	2.9

FCMminiR	1	2.9
FCR	3	8.6
R, CR	1	2.9
Steroids	3	8.6

Treatment regimens of the specific number of patients within our cohort. When patients have received several treatments, these are separated by a comma, e.g. CLB, F denoted chlorambucil followed by fludarabine. Abbreviations: Benda, Bendamustine; C, cyclophosphamide; CLB, chlorambucil; F, fludarabine; FC, fludarabine + cyclophosphamide; FCR, fludarabine + cyclophosphamide + rituximab; CLB+pred, Chlorambucil + Prednisone; R-CHOP, Rituximab + Cyclophosphamide, doxorubicin Hydrochloride, vincristine (Oncovin), Prednisone; HDMP, high-dose methylprednisone; FCMminiR, Fludarabine + Cyclophosphamide + Mitoxantrone with lower dose Rituximab.

Table S2. (**A**) The number of patients who had "high" and "low" BCL6 and BACH2 RNA and protein expression, (**B**) the number of patients in each molecular group separated as "high" and "low" RNA expression level based on ROC analyses.

**A**					
**Characteristics, *n* = 102**	***N***	**%**	**Characteristics, *n* = 91**	***N***	**%**
***BACH2* RNA Expression**			**BACH2 protein Expression**		
High	57	56.0	High	41	45.0
Low	45	44.0	Low	50	55.0
***BCL6* RNA Expression**			**BCL6 protein Expression**		
High	68	66.7	High	36	39.6
Low	34	33.3	Low	55	60.4

**B**						
**Cytogenetics *n* = 101**	***N***	**%**	***BACH2* High**	***N***	***BCL6* High**	**%**
13q	62	61.4	35	56.5	42	67.7
11q	20	19.8	12	60.0	14	70.0
12+	6	5.9	2	33.3	4	66.7
17p	8	7.9	3	37.5	4	50.0
Normal karyotype	24	23.8	12	50.0	15	62.5

Table S3. (A) Correlation analysis between *BACH2* relative quantity (RQ) and the most common CLL prognosticators, (B) Correlation analysis between *BCL6* RQ and the most common CLL prognosticators, (C) Univariate and multivariate analysis for overall survival.

**A**									
		**13q del**	**11q del**	**Trisomy 12**	**17p del**	**ZAP70 status**	**CD38 status**	**IGHV status**	**TP53 mutation**
**RQ BACH2**	**R**	0.03	0.05	-0.11	-0.11	-0.33	-0.42	-0.14	-0.04
	p	0.80	0.65	0.27	0.29	0.17	0.01	0.28	0.71
	N	101.00	101.00	101.00	101.00	19.00	36.00	63.00	100.00

**B**									
		**13q del**	**11q del**	**trisomy 12**	**17p del**	**ZAP70 status**	**CD38 status**	**IGHV status**	**TP53 mutation**
**RQ BCL6**	**R**	0.04	0.04	0.00	-0.10	-0.19	0.00	0.02	-0.13
	p	0.71	0.70	0.99	0.31	0.43	1.00	0.87	0.22
	N	101.00	101.00	101.00	101.00	19.00	36.00	63.00	100.00

**C**		
	**HR (95% CI)**	***p*-value**
**i) Univariate Model**		

BACH2 RQ	0.996(0.993–0.998)	0.003
Age	1.050(1.010–1.091)	0.014
17p deletion	3.681(1.272–10.650)	0.016
**ii) Multivariate Model**		
BACH2 RQ	0.995(0.992–0.998)	0.007
Age	1.055(1.016–1.095)	0.005
17p deletion	3.438(1.127–10.492)	0.030

Table S4. Univariate (A) and multivariate (B and C) analyses when all the covariates are considered in the same model for RNA expression level of *BACH2* and *BCL6*.

	**A**	
**Univariate**	**HR (95% CI)**	*p*-value
IgVH	3.093(1.362–7.020)	0.007
	**B**	
Multivariate	HR	*p*-value
IgVH	5.530(2.193–13.940)	<0.001
BACH2	0.996(0.992–1.00)	0.071
Age	1.099(1.045–1.154)	<0.001
17p	2.418(0.700–8.352)	0.163
	**C**	
Multivariate	HR	*p*-value
IgVH	5.946(2.355–15.012)	<0.001
BACH2	0.996(0.992–0.999)	0.049
Age	1.10(1.048–1.160)	<0.001

Table S5. Multivariate analyses when all the covariates are considered in the same model for treatment status and protein expression levels of BACH2 and BCL6.

**Multivariate**	**HR**	***p*-value**
BACH2 protein expression	0.995(0.992–0.999)	0.005
Age	1.058(1.017–1.101)	0.005
17p	3.183(1.037–9.764)	0.043
BACH2 protein expression	0.994(0.991–0.998)	0.002
Age	1.057(1.013–1.103)	0.011
17p	2.220(0.625–7.882)	0.217
BCL6 protein expression	0.962 (0.794–1.165)	0.691
BACH2 protein expression	0.996(0.993–0.999)	0.018
Age	1.069(1.022–1.118)	0.003
17p	3.242(1.046–10.050)	0.042
BACH2 protein expression	0.439(0.185–1.039)	0.061
BACH2 protein expression	0.996(0.993–0.999)	0.018
Age	1.07(1.020–1.115)	0.004
17p status	3.33(1.067–10.373)	0.038
BCL6 protein expression	0.760(0.250–2.307)	0.628
Treatment Status	0.780(0.354–1.717)	0.537

Table S6. Proteins co-immunoprecipitated with BACH2 or BCL6 detected from the co-immunoprecipitation and subsequent MS studies.

**Proteins co-immunoprecipitated with BACH2 or BCL6**	**Name and Function**
Q9NTK5	Obg like ATPase 1
O43166	Signal induced proliferation associated 1 like 1

Q8WW12	PEST proteolytic signal containing nuclear protein
P18084	Integrin subunit beta 5
Q6ZMT4	Lysine demethylase 7A
P00403	Mitochondrially encoded cytochrome c oxidase II
P53618	Coatomer protein complex subunit beta
P17174	Glutamic-oxaloacetic transaminase 1
Q99832	Chaperonin containing TCP1 subunit 7
P16885	Phospholipase C gamma 2
Q5RKV6	Exosome component 6
P49321	Nuclear autoantigenic sperm protein
P55036	Proteasome 26S subunit, non-ATPase 4
P04843	Ribophorin I
P00505	Glutamic-oxaloacetic transaminase 2
P05455	Sjogren syndrome antigen B
Q9Y5A9	YTH N6-methyladenosine RNA binding protein 2
P25786	Proteasome subunit alpha 1
Q14697	Glucosidase II alpha subunit
Q15181	Pyrophosphatase (inorganic) 1
P61221	ATP binding cassette subfamily E member 1
Q9Y285	Phenylalanyl-tRNA synthetase alpha subunit
O43809	Nudix hydrolase 21
Q14204	Dynein cytoplasmic 1 heavy chain 1
Q9H9B4	Sideroflexin 1
Q15046	Lysyl-tRNA synthetase
P08758	Annexin A5
P54819	Adenylate kinase 2
P50991	Chaperonin containing TCP1 subunit 4
P30041	Peroxiredoxin 6
O75347	Tubulin folding cofactor A
Q99798	Aconitase 2
O76094	Signal recognition particle 72
P37837	Transaldolase 1
Q13428	Treacle ribosome biogenesis factor 1
P58505	Chromosome 21 open reading frame 58
Q13011	Enoyl-CoA hydratase 1
P52566	Rho GDP dissociation inhibitor beta
Q96RU3	Formin binding protein 1
P54727	RAD23 homolog B, nucleotide excision repair protein
Q96RP9	G elongation factor mitochondrial 1
P24539	ATP synthase, H+ transporting, mitochondrial Fo complex subunit B1
Q86UV5	Ubiquitin specific peptidase 48
Q9Y4P3	Transducin beta like 2
P07355	Annexin A2
Q9P2E9	Ribosome binding protein 1
Q96AE4	Far upstream element binding protein 1
P30405	Peptidylprolyl isomerase F
P62495	Eukaryotic translation termination factor 1
P49368	Chaperonin containing TCP1 subunit 3

Table S7. Proteins and their function that exclusively associated with BACH2 and BCL6 detected from the co-immunoprecipitation and subsequent MS studies.

BACH2	Name	Protein function	BCL6	Name	Protein function
Q99460	PSMD1	Component of 26S proteasome, a multiprotein complex involved in the ATP-dependent degradation of ubiquitinated proteins.	Q8WYA6	CTNNBL1	Beta-catenin-like protein 1.
P84095	RHOG	RAS homolog family member G, Rho-related GTP-binding protein.	Q5VYK3	KIAA0368	Proteasome adapter and scaffold protein ECM29.
Q6NUM9	RETSAT	All-trans-retinol 13,14-reductase.	P61204	ARF3	ADP-ribosylation factor 3.
P62263	RPS14	40S ribosomal protein S14.	Q15833	STXBP2	Syntaxin-binding protein 2.
Q86V59	PNMA8A	Paraneoplastic antigen-like protein 8A.	Q99623	PHB2	Prohibitin 2.
P02533	KRT14	Keratin, type I cytoskeletal 14.	P07602	PSAP	Prosaposin.
P15924	DSP	Desmoplakin, major high molecular weight protein of desmosomes.	P25205	MCM3	DNA replication licensing factor MCM3.
Q15020	SART3	Squamous cell carcinoma antigen recognized by T-cells 3.	P33991	MCM4	DNA replication licensing factor MCM4.
O00487	PSMD14	26S proteasome non-ATPase regulatory subunit 14.	O75553	DAB1	Disabled homolog 1.
Q96A19	CCDC102A	Coiled-coil domain containing 102A.			

Table S8. Gene Ontology (GO) term analyses of proteins interacting with both BACH2 and BCL6 in biological process and cellular component.

References

1. Wiśniewski, J.R.; Gaugaz, F.Z. Fast and Sensitive Total Protein and Peptide Assays for Proteomic Analysis. Analytical Chemistry. **2015**, *87*, 4110–4116.
2. Wiśniewski, J.R.; Duś, K.; Mann, M. Proteomic workflow for analysis of archival formalin-fixed and paraffin-embedded clinical samples to a depth of 10 000 proteins. PROTEOMICS – Clinical Applications. **2013**, *7*, 225–233.
3. Gawin, M.; Wojakowska, A.; Pietrowska, M.; Marczak, Ł.; Chekan, M.; Jelonek, K.; et al. Proteome profiles of different types of thyroid cancers. Molecular and Cellular Endocrinology. **2018**, *472*, 68–79.
4. Mi, H.; Thomas, P. PANTHER pathway: an ontology-based pathway database coupled with data analysis tools. Methods in molecular biology (Clifton, NJ). **2009**, *563*, 123–140.
5. Jensen, L.J.; Kuhn, M.; Stark, M.; Chaffron, S.; Creevey, C.; Muller, J.; et al. STRING 8—a global view on proteins and their functional interactions in 630 organisms. Nucleic Acids Research. **2008**, *37*, D412-D416.
